# Biometric Identification Method for Heart Sound Based on Multimodal Multiscale Dispersion Entropy

**DOI:** 10.3390/e22020238

**Published:** 2020-02-20

**Authors:** Xiefeng Cheng, Pengfei Wang, Chenjun She

**Affiliations:** 1 College of Electronic and Optical Engineering & College of Microelectronics, Nanjing University of Posts and Telecommunications, Nanjing 210023, China; chengxf@njupt.edu.cn (X.C.); 1015020702@njupt.edu.cn (C.S.); 2 National and Local Joint Engineering Laboratory of RF Integration & Micro-Assembly Technology, Nanjing 210003, China

**Keywords:** heart sound, ICEEMDAN, RCMDE, Fisher ratio, biometric characterization

## Abstract

In this paper, a new method of biometric characterization of heart sounds based on multimodal multiscale dispersion entropy is proposed. Firstly, the heart sound is periodically segmented, and then each single-cycle heart sound is decomposed into a group of intrinsic mode functions (IMFs) by improved complete ensemble empirical mode decomposition with adaptive noise (ICEEMDAN). These IMFs are then segmented to a series of frames, which is used to calculate the refine composite multiscale dispersion entropy (RCMDE) as the characteristic representation of heart sound. In the simulation experiments I, carried out on the open heart sounds database Michigan, Washington and Littman, the feature representation method was combined with the heart sound segmentation method based on logistic regression (LR) and hidden semi-Markov models (HSMM), and feature selection was performed through the Fisher ratio (FR). Finally, the Euclidean distance (ED) and the close principle are used for matching and identification, and the recognition accuracy rate was 96.08%. To improve the practical application value of this method, the proposed method was applied to 80 heart sounds database constructed by 40 volunteer heart sounds to discuss the effect of single-cycle heart sounds with different starting positions on performance in experiment II. The experimental results show that the single-cycle heart sound with the starting position of the start of the first heart sound (S1) has the highest recognition rate of 97.5%. In summary, the proposed method is effective for heart sound biometric recognition.

## 1. Introduction

Heart sound is a complex, non-stationary and quasi-periodic signal that is consisted of multiple heartbeats or cardiac cycles, which mainly contain components such as the first heart sound S1, the second heart sound S2, systolic murmur and diastolic murmur. Heart sound originates from the opening and closing of the heart valve and the turbulence of blood, which contains physiological information, such as atria, ventricles, major vessels, cardiovascular vessels and functional status of various valves, and could reflect mechanical activity and structure status of heart. As the biometric characteristics, the biggest advantage of heart sound is universality, stability, uniqueness and collectability [1]. So far, there have been studies that have verified the feasibility of heart sound signals for biometric identification. The heart sound signal as an option for biometric identification was first introduced by Beritelli and Spadaccini [2]. Their method needs to locate and describe S1 and S2, then chirp-z transform (CZT) is performed to obtain the feature set, and finally, Euclidean distance (ED) is used as classifier. In another study, Phua et al. [3] introduced linear frequency band cepstrum (LFBC) for heart sound feature extraction and used two classifiers of vector quantization (VQ) and Gaussian mixture model (GMM) for classification and recognition. Beritelli and Spadaccini [4] continued improving the performance of phonocardiogram (PCG), building a human recognition system based on 13 MFCC extracted from S1 and S2 for feature extraction and First-to-Second ratio distance (FSR), achieving an equal error rate (EER) of 9% on 50 different people. Beritelli et al. [5] discussed that increasing the test set from 50 to 80 did not give a negative impact on EER. Fatemian et al. [6] Proposed a PCG signal identification and verification system. The system is based on wavelet preprocessing, feature extraction using short-time Fourier transform (STFT), feature dimension reduction using linear discriminant analysis (LDA) and majority voting using Euclidean distance for classification. Tran et al. [7] Used eight feature sets such as temporal shape, spectral shape, Mel-frequency cepstral coefficients (MFCC), linear frequency cepstral coefficients (LFCC), harmonic feature, rhythmic feature, cardiac feature, GMM-super vector as heart sound biometric recognition features, using two feature selection techniques and using support vector machine (SVM) for 52 users Classification recognition. Cheng et al. [8] introduced a human feature extraction method based on an improved circular convolution (ICC) slicing algorithm combined with independent subband function (ISF). The technology uses two recognition steps to obtain different human heart sound characteristics to ensure validity, and then uses similar distances for human heart sound pattern matching. Chen et al. [9] proposed a biometric recognition system based on heart sounds. The system uses wavelet for noise reduction, MFCC for feature extraction and Principal component analysis (PCA) for feature dimension reduction. Zhong et al. [10] proposed a biometric method based on cepstrum coefficients combined with GMM. These cepstrum coefficients are MFCC and LPCC, which are applied to 100 heart sounds of 50 people to test the algorithm. Zhao et al. [11] proposed a heart sound system based on marginal spectrum analysis and the classifier is based on VQ. Babiker et al. [12] present the design of a system for access control using a heart sound biometric signature based on energy percentage in each wavelet coefficients and MFCC feature. Db5 wavelet decomposition is used for noise reduction and ED is used for classification. The results show that the MFCC feature has better performance than the wavelet coefficient energy percentage. Akhter et al. [13] explored the possibility of using heart rate variability (HRV) in biometrics. They designed hardware and software for data collection. They also developed software for HRV analysis in Matlab, which uses various HRV Analysis techniques (such as statistics, spectrum, geometry, etc.) generate 101 HRV parameters (features), and use five different wrapper algorithms for feature selection, and obtain 10 reliable features from the 101 parameters, and finally use K Nearest Neighbor (KNN) classifies objects. The above introduces the common methods of heart sound biometrics. It can be found that the information entropy theory has not been applied in this field. At the same time, the information entropy theory has shown good results in the biological recognition of electrocardiogram (ECG) and electroencephalogram (EEG) signals [14,15,16]. In this paper, for the first time, multiscale entropy theory is introduced for the study of heart sound biometrics.

Ensemble empirical mode decomposition (EEMD) is a widely-used tool for the analysis of biomedical signals. It was proposed to overcome the deficiencies of ending effects and mode mixing in non-stationary signal decomposition when applying Empirical mode decomposition (EMD) to the time series. Recently, a new signal decomposition method based on the EEMD is presented, named as improved complete ensemble empirical mode decomposition with adaptive noise (ICEEMDAN), which provides a better spectral separation of the modes and a lesser number of sifting iterations is needed, reducing the computational cost. In this paper, ICEEMDAN is employed for heart sound signal decomposition to extract effective intrinsic mode functions (IMFs). To quantify the feature information of IMFs extracted from heart sound signals, dispersion entropy (DE), a new measure of uncertainty or irregularity, is introduced. The method tackles sample entropy (SE) and permutation entropy (PE) limitation. As a result of the relevance and the possible usefulness of DE in several signal analyses, it is important to understand the behavior of the technique for various kinds of classical signal concepts such as amplitude, frequency, noise power and signal bandwidth. In addition, the coarse-graining process is introduced to improve DE performance in estimating the complexity at the multiple time scales data, which is named as multiscale dispersion entropy (MDE). Recently, the refined composite MDE (RCMDE) is proposed to improve the computing speed and stability, which is more applicable to process the short and noisy signals in biomedical applications.

**Figure 1:**
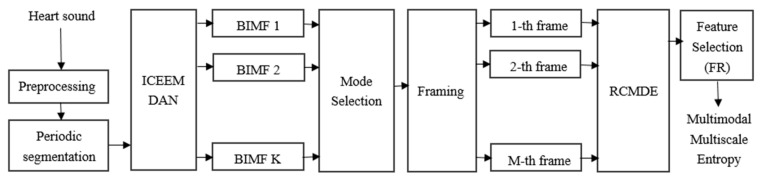
The feature generation flowchart of the multimodal multiscale dispersion entropy.

To avoid the error, the testing data should be consistent with the length of the corresponding training data. Furthermore, the heart sound signal has pseudo-periodicity, and each cardiac cycle contains the dynamic acoustic characteristics of the heart structure. The cardiac cycle is different for each individual, which also reflects physiological characteristics between individuals. Therefore, this work takes single-cycle heart sound as the input of the proposed method, and RCMDE is combined with ICEEMDAN to quantify the important biometric information of the individual contained in every cardiac cycle. For the heart sound signal more than one cycle, it is firstly periodically segmented, and then the single cycle of heart sounds is decomposed into a group of IMFs by ICEEMDAN. These IMFs are then segmented to a series of frames, which is used to calculate the RCMDE as a characteristic representation of the heart sound. In addition, feature selection was performed to remove redundant features through the Fisher ratio (FR), and then ED is used to metric and match the features, and finally forming a new method based on ICEMDAN-RCMDE-FR-ED. At the same time, it can be considered that ICEEMDAN-RCMDE-FR has generated a kind of biometric characterization of heart sounds, which is named as the multimodal multiscale dispersion entropy. The feature generation flowchart of the multimodal multiscale dispersion entropy is as Figure 1.

## 2. Materials and Methods 

### 2.1. Mathematical Model of Heart Sound

In heart sound biometric recognition, heart sound is a non-stationary and quasi-periodic signal due to the rhythmicity of the heartbeat. Although the waveform of each cardiac cycle of heart sound has slight differences in time and magnitude, heart sound can be approximated to the periodic signals in the mathematics model. At the same time, since each cardiac cycle of heart sound contains four main components, including the first heart sound S1, the second heart sound S2, systolic murmur and diastolic murmur, cardiac cycles of heart sound are considered as the main objective of the biometric. The mathematical model of heart sound can be described as follows:

(1) $$\left\{ \begin{array}{c} x(i)=\sum_{n=0}^{N}x_{T}(i-nL)\\ x_{T}(i)=S_{1}(i)+Sysmur(i)+S_{2}(i)+Diasmur(i) \end{array}\right.$$

In the first step of Formula (1), $x_{T}(i),i=1,2,\ldots ,L$, represents any period in the heart sound, the length of heart sound is $L=T*F_{s}$, where T represents the cardiac cycle, $F_{s}$ represents the sampling frequency; x(i),i=1,2,…,N∗L, represents a heart sound signal containing N cardiac cycles. In the second step of Formula (1), $S_{1}(i)$ represents the first heart sound S1, Sysmur(i) represents the systolic murmur, $S_{2}(i)$ represents the second heart sound S2 and Diasmur(i) represents the diastolic murmur.

### 2.2. ICEEMDAN Method

Empirical Mode Decomposition (EMD) is an adaptive method for analyzing non-stationary signals originating from nonlinear systems. It decomposes the original signal into the sum of the intrinsic mode functions (IMFs), which can be described as follows [17]:

(1) Set k=0 and find all extremums of $r_{0}=x$.
(2) Interpolate between the minimum value (the maximum value) of $r_{k}$ to obtain the lower (upper) envelope $e_{\min}$ ($e_{\max}$).
(3) Calculate the average envelope: $\mathrm{me}=(e_{\min}+e_{\max})/2$.
(4) Calculate the candidate IMF: $d_{k+1}{=r}_{k}-\mathrm{me}$.
(5) Is $d_{k+1}$ an IMF?
  Yes, save d_(k+1), calculate the residual $r_{k+1}=x-\sum_{i=1}^{k}d_{i}$, make k=k+1, and put $r_{k}$ as input data in step (2).
  No, $d_{k+1}$ is taken as input data in step (2).
(6) Continue to cycle until the final residual $r_{k}$ meets some predefined stopping criteria.

The improved complete ensemble empirical mode decomposition with the adaptive noise (ICEEMDAN) method has been proved to be suitable for the processing of biomedical signals. The algorithm not only overcomes the mode mixing problem of EMD but also eliminates the spurious mode in CEEMDAN. Let $E_{k}(\cdot )$ denote the operator of the kth modal obtained by EMD, $\omega^{\left(i\right)}$ denotes the realization of white noise with zero mean and unit variance and M(⋅) denotes the operator for calculating the local mean of the signal. The realization steps of ICEEMDAN algorithm are as follows [18,19]:

(1) Calculate the local mean of the signal by I-times realization of EMD: $r_{2}=r_{1}+\beta_{1}E_{2}(\omega^{\left(i\right)})$, get the first residual $r_{1}=\langle M(x^{\left(i\right)})\rangle$, where 〈⋅〉 represents the average operator.
(2) The first modal is calculated from the residual $r_{1}$ obtained in the step (1): ${\tilde{d}}_{1}=x-r_{1}$.
(3) The second residual is calculated by $x^{\left(i\right)}=x+\beta_{0}E_{1}(\omega^{\left(i\right)})$, and defines the second mode: ${\tilde{d}}_{1}=r_{1}-r_{2}=r_{1}-\langle M(r_{1}+\beta_{1}E_{2}(\omega^{\left(i\right)}))\rangle$.
(4) For k=3,4,…,K, calculate the kth residual: $r_{k}=\langle M(r_{k-1}+\beta_{k-1}E_{k}(\omega^{\left(i\right)}))\rangle$.
(5) Calculate the kth modal: ${\tilde{d}}_{k}=r_{k-1}-r_{k}$.
(6) Go to the next k of step (4) until all modes are obtained.

The constant $\beta_{k-1}$ is selected to adjust the signal-to-noise ratio (SNR) between the residual and the added noise. For k=1, $\beta_{0}=\varepsilon_{0}std(x)/std(E_{1}(\omega^{\left(i\right)}))$, where std(⋅) represents the standard deviation, $\varepsilon_{0}$ is the reciprocal of the required SNR between the input signal *x* and the first added noise. For k≥2, $\beta_{k}=\varepsilon_{0}std(r_{k})$.

### 2.3. RCMDE Method

Multiscale dispersion entropy (MDE) is a combination of coarse-grained and dispersion entropy, and the refine composite multiscale dispersion entropy (RCMDE) improves MDE in that the different starting time of the coarse-grained time series corresponding to the different scale factors *τ* is adopted. Based on multiscale techniques, the main steps for calculating RCMDE are as follows [20,21,22]:

(1) The first is to construct a continuous coarse-grained time series. For a univariate signal x(i)(i=1,2,…,N), Its *J*-th coarse-grained time series $x_{J}^{\left(\tau \right)}=\left\{x_{J,1}^{\left(\tau \right)},x_{J,2}^{\left(\tau \right)},\ldots \right\}$ can be showed as follows:

(2) $$x_{J,j}^{\left(\tau \right)}=\frac{1}{\tau }\sum_{i=(j-1)\tau +J}^{j\tau +J-1}x_{i},1\leq j\leq N,1\leq J\leq \tau$$

where τ is the scale factor, and the original time series x is scaled by controlling the value of τ.

(2) Map $x_{J,j}^{\left(\tau \right)}$ into $y_{J,j}^{\left(\tau \right)}$ with the normal cumulative distribution function:

(3) $$y_{J,j}^{\left(\tau \right)}=\frac{1}{\sigma \sqrt{2\pi }}\int_{-\infty }^{x_{J,j}^{\left(\tau \right)}}e^{\frac{-(\tau -\mu^{2})}{2\sigma^{2}}}dt$$

where σ and μ represent the standard deviation and mean of $x_{J,j}^{\left(\tau \right)}$, respectively.

(3) Assign each $y_{J,j}^{\left(\tau \right)}$ to an integer from Label 1 to *c* using a linear algorithm. The mapped signal can be defined as follows:

(4) $$z_{J,j}^{\left(\tau ,c\right)}=round(c\cdot y_{J,j}^{\left(\tau \right)}+0.5)$$

(4) Define embedding vector $z_{J,j}^{\left(\tau ,c,m\right)}$ with embedding dimension m and time delay *d* as:

(5) $$z_{J,j}^{\left(\tau ,c,m\right)}=\{z_{J,j}^{c},z_{J,j+d}^{c},\ldots ,z_{J,j+(m-1)d}^{c}\}$$

Each time series $z_{J,j}^{\left(\tau ,c,m\right)}$ is mapped to a dispersion pattern $\pi_{v_{0}v_{1}\ldots v_{m-1}}$, where:

$$z_{J,j}^{\left(\tau ,c\right)}=v_{0},z_{J,j+d}^{\left(\tau ,c\right)}=v_{1},\ldots ,z_{J,j+(m-1)d}^{\left(\tau ,c\right)}=v_{m-1}$$

(5) For each dispersion pattern, the relative frequency can be obtained as:

(6) $$p(\pi_{v_{0}v_{1}\ldots v_{m-1}})=\frac{Number\{j|j\leq N-(m-1)d,z_{J,j}^{\left(\tau ,c,m\right)}has\_type\_v_{0}v_{1}\ldots v_{m-1}\}}{N-(m-1)d}$$

where $p(\pi_{v_{0}v_{1}\ldots v_{m-1}})$ represent the number of dispersion pattern which is assigned to $z_{J,j}^{\left(\tau ,c,m\right)}$ divided by the total number of embedding signals with embedding dimension m.

(6) Based on Shannon’s definition of entropy, multiscale dispersion entropy with embedding dimension *m*, time delay *d*, and the number of classes *c* can be defined as:

(7) $$RCMDE(x,m,c,d,\tau )=-\sum_{\pi =1}^{c^{m}}p(\pi_{v_{0}v_{1}\ldots v_{m-1}})Inp(\pi_{v_{0}v_{1}\ldots v_{m-1}})$$

### 2.4. Feature Selection

Fisher Ratio (FR) is proposed on the basis of Fisher criterion. It is used to measure the classification and recognition ability of features and has been successfully used by Pruzansky and Mathews in the research of speech recognition [23]. In this paper, the Fisher ratio is used to select the optimal features and the steps are as follows:

(1) Calculate the inter-class dispersion $\sigma_{\mathrm{between}}$, which is used to measure the degree of dispersion of the r-dimensional feature parameters between the heart sound signals of various categories. The calculation formula is:

(8) $$\sigma_{\mathrm{between}}=\sum_{i=1}^{M}{\left(\mu_{r}^{\left(i\right)}-\mu_{r}\right)}^{2}$$

where *M* represents the total number of heart sound samples, $\mu_{r}^{\left(i\right)}$ is the mean value of the *r*-dimensional feature parameters of the *i*-th type heart sound signal, and $\mu_{r}$ is the mean value of the r-dimensional feature parameters in all heart sound signals.

(2) Calculate the intra-class dispersion $\sigma_{\mathrm{within}}$, which is used to measure the degree of dispersion in the r-dimensional feature parameters of a certain type of heart sound signal. The calculation formula is:

(9) $$\sigma_{\mathrm{within}}=\sum_{i=1}^{M}\left[\frac{1}{n_{i}}\sum_{j=1}^{n_{i}}{\left(x_{r}^{\left(j\right)}-\mu_{r}^{\left(i\right)}\right)}^{2}\right]$$

where $n_{i}$ is the number of heart sound samples of the *i*-th type heart sound signal, $x_{r}^{\left(j\right)}$ is the r-dimensional feature parameter of the *j*-th heart sound sample of the *i*-th heart sound signal.

(3) To calculate the Fisher ratio, the calculation formula is:

(10) $$F_{r}=\frac{\sigma_{between}}{\sigma_{within}}$$

where $F_{r}$ is the Fisher ratio of the r-dimensional characteristic parameter.

(4) Sort the Fisher ratio of each dimension feature parameter in descending order:

(11) $$F_{1},F_{2},\ldots ,F_{R}\to F_{r_{1}}>F_{r_{2}}>\ldots >F_{r_{R}}$$

where $r_{i}\in \{1,2,\ldots ,R\},i=1,2,\ldots ,R$, R is the dimension of the characteristic parameter.

(5) The larger the Fisher ratio, the stronger the classification and recognition ability of the feature parameter of the dimension. According to this principle, the top $N_{r}$ dimensional feature parameters ranked first in (4) is selected as the optimal features.

### 2.5. Matching Recognition

This paper adopts the Euclidean distance (ED) and the close principle to realize the pattern recognition of the user’s heart sound. The idea of the algorithm is as follows: when the data and labels in the training set are known, compare the one-dimensional feature vector of the test data with the corresponding feature vector in the training set to find the one-dimensional feature vector most similar to it in the training set, then the category corresponding to the test feature vector is the category corresponding to the training feature vector. The algorithm steps are:

(1) Calculate the distance between the test data and each training data;

For the feature vector v of the test data and the feature vector $v_{A}$ of the A-th training data in the heart sound database, the Euclidean distance $d_{A}$ in the D dimension Euclidean space is calculated as follows:

(12) $$d_{A}=\sqrt{\sum_{i=1}^{D}{\left(v(i)-v_{A}(i)\right)}^{2}}$$

where A=1,2,…,C, C is the number of training data, and *D* is the dimension of the feature vector.

(2) Sort in increasing order of distance:

(13) $$d_{1},d_{2},\ldots ,d_{C}\to d_{x_{1}}>d_{x_{2}}>\ldots >d_{x_{C}}$$

where $x_{i}\in \{1,2,\ldots ,C\},i=1,2,\ldots ,C$.

(3) According to the selection principle, the closer the distance, the higher the degree of matching between the two data. The category corresponding to the closest feature vector $v_{x_{1}}$ is selected as the prediction classification of the test data.

### 2.6. Evaluation Methods

This paper uses the following three indicators to evaluate the proposed algorithm [24]:

(1) Average test accuracy: The $\bar{CRR}$ obtained by averaging the CRR of J experiments was used as the final experimental result, as shown in Equation (9). Considering the calculation amount and accuracy comprehensively, *J* = 200 is taken in the following experiment of parameter selection and algorithm comparison.

(14) $$\left\{ \begin{array}{c} CRR(j)=\frac{Number\_of\_correctly\_identified\_subjects\_in\_trial\_j}{Total\_number\_of\_subjects}\\ \bar{CRR}=\frac{1}{J}\sum_{j=1}^{J}CRR(j) \end{array}\right.$$

(2) Kappa coefficient: Kappa coefficient is an index to measure the accuracy of multi-classification. Its calculation formula is as follows:

(15) $$Kappa=\frac{p_{0}-p_{e}}{1-p_{e}}$$

Among them, $p_{0}$ is the sum of the number of correctly classified samples of each class divided by the total number of samples, which is the overall classification accuracy. Suppose the number of true samples in each class is $a_{1},a_{2},\ldots ,a_{c}$, and the number of predicted samples in each class is $b_{1},b_{2},\ldots ,b_{c}$, and the total number of samples is num.

(16) $$p_{e}=\frac{a_{1}b_{1}+a_{2}b_{2}+\ldots +a_{c}b_{c}}{num\cdot num}$$

The kappa coefficient is usually between 0 and 1 and can be divided into five groups to represent different levels of classification accuracy: 0.0 to 0.20 extremely low classification accuracy, 0.21 to 0.40 general classification accuracy, 0.41 to 0.60 high classification accuracy, 0.61–0.80 very high classification accuracy and 0.81–1 extremely high classification accuracy.

(3) *t*-test: *t*-test uses the t-distribution theory to infer the probability of a difference occurring, thereby comparing whether the difference between the two averages is significant. This paper is repeatedly training/testing by randomly dividing the training set/test set multiple times, therefore this will get multiple test accuracy rates. Therefore, the *t* test can be used to verify whether the ${\bar{CRR}}_{200}$ selected in this paper can be used as generalization Accuracy. Assuming the generalization accuracy $\mu_{0}={\bar{CRR}}_{200}$, we get *n* test accuracy rates, CRR(i),i=1,2,…,n, then the average test accuracy μ and variance $\sigma^{2}$ are:

(17) $$\mu =\frac{1}{n}\sum_{i=1}^{n}CRR(i)$$

(18) $$\sigma^{2}=\frac{1}{n-1}\sum_{i=1}^{n}{\left(CRR(i)-\mu \right)}^{2}$$

Considering that the accuracy of these n tests can be regarded as independent sampling of the generalization accuracy $\mu_{0}$, then the variable $t=\frac{\sqrt{n}(\mu -\mu_{0})}{\sigma }$ follows a *t*-distribution with *n*−1 degrees of freedom. This paper uses the following *t*-test steps:

(1) First establish hypotheses and determine the test level α:

$H_{0}:\mu =\mu_{0}$ (zero hypothesis), $H_{1}:\mu \neq \mu_{0}$ (alternative hypothesis), using bilateral hypothesis, α commonly used values are 0.05 and 0.1, the test level is α=0.05 in this paper.

(2) Calculate the test statistics: $t=\frac{\sqrt{n}(\mu -\mu_{0})}{\sigma }$.

(3) Check the corresponding critical value table to determine the critical value $t_{\left(\alpha ,n\right)}$ and conclude: If the value of t is within the critical value range $\left[-t_{\left(\alpha ,n\right)},t_{\left(\alpha ,n\right)}\right]$, you cannot reject the assumption that $H_{0}:\mu =\mu_{0}$, you can think that the generalization accuracy is μ, the degree of confidence is 1−α; otherwise, the hypothesis can be rejected, that is, under this significance degree, the generalization accuracy $\mu_{0}$ can be considered to be significantly different from the average test accuracy μ.

## 3. Results and Discussion

### 3.1. Data Sources

**Figure 2:**
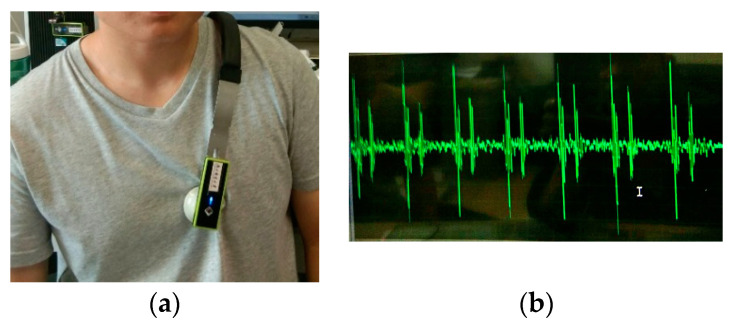
Heart sound database collected by our group: (**a**) Ω shoulder-belt wireless heart sound sensor; (**b**) The processing of collecting heart sound.

To verify the effectiveness of the proposed method, the open databases of heart sound recordings and the heart sound database built by our research group are both analyzed. The open database used in this paper consists of 72 heart sounds from the three open heart sound databases Michigan, Washington and Littman, including 18 normal heart sounds and 54 abnormal heart sounds. Among them, 23 cases and 16 heart sounds were obtained from the Michigan and Washington heart sound database, and 33 heart sounds were selected from 3M’s Littman heart sound database, because 3 heart sounds that did not meet the experimental conditions (i.e., not satisfied should contain at least two cardiac cycles) was abandoned. For the Michigan and Washington heart sound databases [25,26], the sampling frequency is 44.1 kHz, and the acquisition time is about 60 s, respectively including 23 and 16 heart sounds. For the Littman heart sound database [27], the sampling frequency is 11.025 kHz, and the acquisition time is about 3 s. The heart sound database built by our research group consisted of 80 cases of heart sound recordings from college student and teacher volunteers, which are collected by using the Ω shoulder-belt wireless heart sound sensor self-developed by our research group (patent number: 201310454575.6) with sampling frequency of 11,025 Hz. Every volunteer is recorded twice at least one-hour intervals, and every time keep approximately 5 s by properly contacting it with the skin of the front chest wall of the subject, as shown in Figure 2. The heart sound recording is from the apex located slightly inside the midline of the left intercostal bone of the fifth intercostal space obtained from the valve area. In addition, the heart sound recordings obtained from the subjects are collected in their calm state, and the recorded heart sound recordings are stored in a .wav format.

### 3.2. Feature Extraction and Recognition

#### 3.2.1. Pretreatment

The original signal is first preprocessed before performing feature extraction and matching recognition. The preprocessing module includes set the labels for heart sounds, downsampling, denoising, and cycle segmentation. Firstly, the 72 heart sound recordings of the three open heart sound databases are set the labels of 1–72 separately to distinguish the individual corresponding to each heart sound. Then the downsampling frequency is set to 2000 Hz, and the background noise when collecting heart sounds is eliminated by using the wavelet packet multi-threshold denoising method. Wavelet packet multi-threshold denoising is through setting a certain threshold value for each layer of wavelet packet coefficients to quantify and analyze each wavelet coefficient, retain useful data and eliminate unnecessary data. Different wavelets may cause different denoising effects, therefore, Biorthogonal HS wavelets developed for heart sound signals [28] is used here to filter in this work. The specific process is as follows:

(1) Performing four-layer HS wavelet packet transform on the noisy signal, and obtain a set of wavelet packet coefficients $wpt_{i},i=1,2,\ldots ,16$;

(2) To quantify the threshold of $wpt_{i}$ separately by selecting the Heursure function, and use the threshold to remove the useless data in $wpt_{i}$;

(3) To perform discrete wavelet reconstruction by using the denoised coefficient $wpt_{i}$, and the reconstructed signal is the denoised signal.

#### 3.2.2. Periodic Segmentation

**Figure 3:**
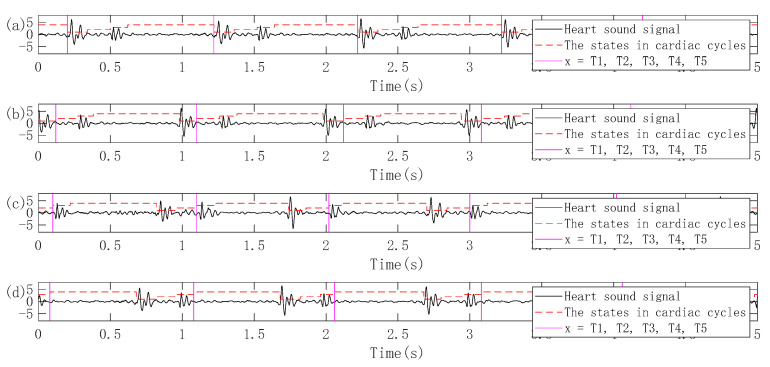
Four methods of heart sound cycle segmentation. (**a**) A series of cardiac cycles segmented from the beginning of S1 to the beginning of the next S1 of the current heart sound recording; (**b**) a series of cardiac cycles segmented from the beginning of the systole to the beginning of the next systole of the current heart sound recording; (**c**) a series of cardiac cycles segmented from the beginning of S2 to the beginning of the next S2 of the current heart sound recording; (**d**) a series of cardiac cycles segmented from the beginning of the diastole to the beginning of the next diastole of the current heart sound recording.

Since the proposed feature extraction method is based on single-period heart sounds, the logical regression (LR) and hidden semi-Markov model (HSMM) heart sound segmentation method proposed by Springer et al. is used in this work, which has proven in the 2016 PhysioNet/CinC Challenge to accurately segment heart sounds in noisy real heart sound recordings with good performance [29,30,31]. In this paper, the heart sound segmentation method is firstly used to assign four states such as S1 (the first heart sound), systole, S2 (the second heart sound) and diastole for the preprocessed heart sound recordings. The time point of the first jump from the initial state of the current heart sound recording to the next state is used as the initial split point. The following four situations may be obtained: (1) a series of cardiac cycles segmented from the beginning of S1 to the beginning of the next S1 of the current heart sound recording; (2) a series of cardiac cycles segmented from the beginning of the systole to the beginning of the next systole of the current heart sound recording; (3) a series of cardiac cycles segmented from the beginning of S2 to the beginning of the next S2 of the current heart sound recording; (4) a series of cardiac cycles segmented from the beginning of the diastole to the beginning of the next diastole of the current heart sound recording. The schematic diagram of the heart sound cycle segmentation corresponding to these four cases is shown in Figure 3.

By the above segmentation method, 72 heart sound recordings in the open heart sound databases are divided into 2005 single-cycle heart sounds, where each heart sound recording is divided into 2–101 single-cycle heart sounds according to their length. In each of the following experiments, a single-period heart sound was randomly selected from the single-cycle heart sounds from the same heart sound recording as a test data, so that the test data contained 72 single-period heart sounds from different individuals, and the remaining 1933 single-cycle heart sounds were used as training data.

#### 3.2.3. Framing and Windowing

Similar to the speech signal, heart sound is also a non-stationary and time-varying signal. Therefore, the heart sound signal is divided into a set of frames to analyze its characteristic parameters. For each frame, the length of the frame is called the frame length. The standard speech frame length is 20 ms to 25 ms, which is not suitable for heart sounds due to its pseudo-periodicity. Reference [32] thinks that the frame length of heart sounds should be longer than 20–25 ms, and it is best when the frame length equals to 256 ms. In this paper, the frame length of the heart sound should be related to the cardiac cycle, and frame lengths should be set different values according to the cardiac cycle. Further, the distance from the start of the frame to the start of the subsequent frame is called the frameshift. To smoothly change the feature parameters, a part of the overlap between adjacent frames is often provided in the case of framing. To prevent spectrum leakage, windowing is usually performed for each frame of heart sounds, usually a Hanning window or a Hamming window.

**Figure 4:**
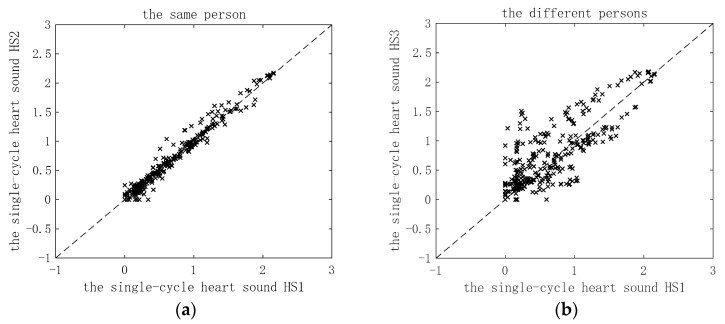
Comparison of refine composite multiscale dispersion entropy (RCMDE) characteristics of single-cycle heart sounds after windowing and framing: (**a**) Comparison of RCMDE characteristics of two single-cycle heart sounds of the same person; (**b**) comparison of RCMDE characteristics of two single-cycle heart sounds of different persons.

The single-cycle heart sound obtained after preprocessing and period segmentation is framed by overlap windowing, and then the RCMDE features of each frame are calculated. Then, the RCMDE features of each frame of the single-cycle heart sound are combined into a one-dimensional feature vector. When calculating RCMDE, four important parameters in RCMDE that may have a greater impact on the results, namely scale factor *τ*, categories *c*, embedding dimension *r* and delay time *τ*. In this experiment, a large number of experiments show when the scaling factor *τ* = 20, the categories *c* = 3, the embedding dimension *m* = 2 and the delay time *d* = 1, the algorithm performance is the best. In Figure 4a, the RCMDE characteristics of two different single-cycle heart sounds of the same person after windowing and framing are compared. As can be seen from the figure, all feature points of the two single-cycle heart sounds are distributed near the 45° line. It shows that the two single-cycle heart sounds are close in their corresponding eigenvalues, and they are relatively matched. In Figure 4b, the RCMDE characteristics of two single-cycle heart sounds of different people after windowing and framing are compared. It can be seen that the two single-cycle heart sounds have more feature points distributed farther from the 45° line, which indicates that the two single-cycle heart sounds have relatively large differences in corresponding feature values, and are not well matched. In Figure 4, the frame length is taken as T/4, the frameshift is taken as T/8 and the Hanning window is used.

From the above analysis, it can be known that the RCMDE feature of single-cycle heart sounds after windowing and framing is feasible for the identification of different individuals. The effect of setting different frame lengths and frameshifts on the performance of the algorithm based on the cardiac cycle is discussed below. Here, adopting the control variable method, the above-mentioned parameters remain unchanged. It is discussed that the frame length takes win = T/*i* (*i* = 1, 2, …, 20) respectively in the condition of no frame overlap, and the corresponding $\bar{CRR}$ is as shown in the left half of Table 1. The result shows that the optimum frame length is T/4. Then, it is discussed that when the frame length takes T/4 unchanged, the frameshift takes inc = win/*i* (*i* = 1, 2, …, 10) respectively, and the corresponding $\bar{CRR}$ is as shown in the right half of Table 1. The result shows that when the frameshift is win/5, the best performance is achieved, and adding the frame overlap latter has not improved $\bar{CRR}$.

#### 3.2.4. ICEEMDAN-RCMDE-FR-ED Algorithm

To achieve a higher $\bar{CRR}$, the ICEMDAN algorithm is used to decompose the training/test cycles into a group of IMFs, and then the hamming window with the window size of T/4 and the window shifting of T/20 is used to frame these IMFs. The training/test IMFs are segmented separately, and each of the obtained heart frames is subjected to RCMDE calculations. The result is sent to the ED algorithm, and the obtained $\bar{CRR}$ is shown in Table 2. Here, the parameters of the ICEEMDAN algorithm are selected as follows, the noise standard deviation is Nstd = 0.2, the number of EMD implementations is NR = 100, the maximum number of screening iterations allowed is MaxIter = 5000, and the SNRFlag = 1 indicates that the signal-to-noise ratio (SNR) is incremental with EMD implementation. Since ICEEMDAN is an adaptive decomposition algorithm, the number of modals obtained from different heart sound cycles may be different. For comparison, only the least number of IMFs obtained by the ICEEMDAN from the heart sound database is shown here.

**Figure 5:**
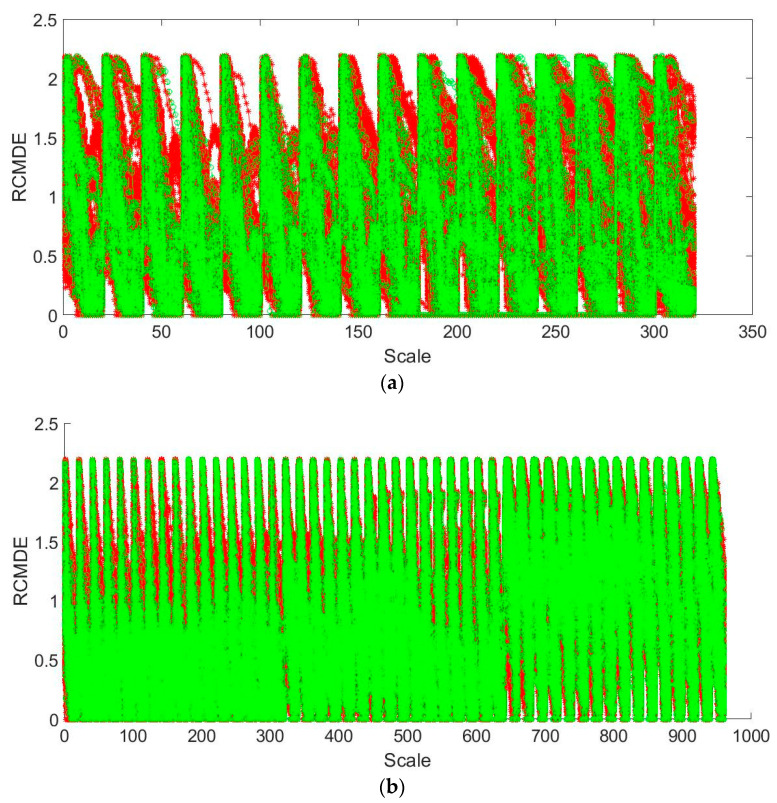
The feature characterization based on the different algorithms (**a**) the feature characterization based RCMDE; (**b**) the new feature characterization based on the combination of improved complete ensemble empirical mode decomposition with adaptive noise (ICEEMDAN) and RCMDE.

It is found through experiments that the first three IMFs of the heart sound cycle, respectively used as the algorithm input, can obtain a higher $\bar{CRR}$ compared with the others. It shows that the first three IMFs not only contain the majority of the information in the heart sound cycle but also dig deep the information in the entire heart sound cycle, which is expressed in a more detailed way. Therefore, adding the features from the above three IMFs to one feature vector as a new heart sound feature is considered. The original heart sound feature representation is shown in Figure 5a. The new heart sound feature representation is shown in Figure 5b. The red dot in the figure represents the single-cycle feature as the training, and the green dot represents the single-cycle feature as the testing. Since the single-cycle heart sounds as the training is much more than the single-cycle heart sounds as the testing, it is shown in the figure below that the green dot is wrapped by the red dot.

It can be found from Figure 5 that the merged features have twice as many feature dimensions as the original features and have great redundancy. Therefore, the Fisher ratio (FR) is used for feature selection. After the features are ranked according to the Fisher ratio, the features are selected. The first $N_{r}$ feature dimensions are used as new heart sound features. After experimental verification, when $N_{r}=300$, the recognition performance is optimal. The ${\bar{CRR}}_{200}$ and Kappa coefficients of respectively using the original heart sound feature and the new heart sound feature with ED and the close principle are shown in Table 3.

It can be seen from Table 3 that the ${\bar{CRR}}_{200}$ and Kappa coefficients on the three public heart sound databases obtained from the feature extraction method based on ICEEMDAN-RCMDE-FR are higher than the feature extraction method based on RCMDE, and can achieve an average recognition rate of 96.08%. The Kappa coefficient is between 0.8 and 1, which indicates that the classification accuracy is extremely high. The following t-test is used to verify whether ${\bar{CRR}}_{200}=96.08\%$ obtained in the above table can be regarded as the generalization accuracy. Here, n random experiments are performed, where *n* = 10, 20, 30, 50, 100, 200, 300, 400, 500, 600, respectively, the average test accuracy μ and standard deviation σ corresponding to n experiments are shown in the left half of Table 4. Here it is assumed that the generalization accuracy $\mu_{0}={\bar{CRR}}_{600}$, the test level α is 0.05 and then the t value of n experiments is obtained according to the t-test steps in Section 2.6, and the corresponding critical value range is also given.

It can be seen from Table 4 that the t values corresponding to n experiments are all within the critical value range $\left[-t_{\left(\alpha ,n\right)},t_{\left(\alpha ,n\right)}\right]$, and the average test accuracy μ corresponding to n experiments can be considered Both can be regarded as generalization accuracy of $\mu_{0}$ and the degree of confidence is 0.95. It can also be found from Table 4 that when the number of experiments n is greater than 200, the average test accuracy μ has basically stabilized at 96.08% and the t value has basically stabilized near 0. Therefore, considering the stability and calculation cost, the number of experiments is taken as *J* = 200, the best generalization accuracy is $\mu_{0}=96.08\%$.

In summary, the feature extraction method based on ICEEMDAN-RCMDE-FR proposed in this paper can achieve a generalization accuracy of 96.08% on three public heart sound databases with a confidence level of 0.95, which shows that the multimodal multiscale dispersion entropy generated by the ICEEMDAN-RCMDE-FR algorithm has good characterization of heart sounds, and it is suitable for the field of biometrics. Considering that the classifier currently used is rough, this may be one of the reasons why the $\bar{CRR}$ cannot be further improved. Therefore, different classifiers such as SVM and KNN are compared with ED, and the results are shown in Table 5. The SVM classifier used here is parameter-tuned. The two main penalty parameters *c* and the kernel function parameter *g* are 64 and 0.001, and the nearest neighbors of the KNN classifier are taken as *k* = 5, 3, 2, respectively. From the results in Table 5, the difference between the best performance of the three classifiers is within 1%. It can be found that the smaller the parameter k of KNN is, the higher the CRR is. When k = 1 or 2, KNN is the ED classifier. The heart sound recordings in the open database are different in length, therefore the data distribution in the single-cycle heart sound database generated by the segmentation is not balanced, which may be the reason that the classifier performance cannot be further improved. Since the ED classifier is relatively simpler, the matching recognition time is also faster. Considering the combination, the ED classifier is most suitable for the heart sound database.

### 3.3. Practical Application of ICEEMDAN-RCMDE-FR-ED Algorithm

**Figure 6:**
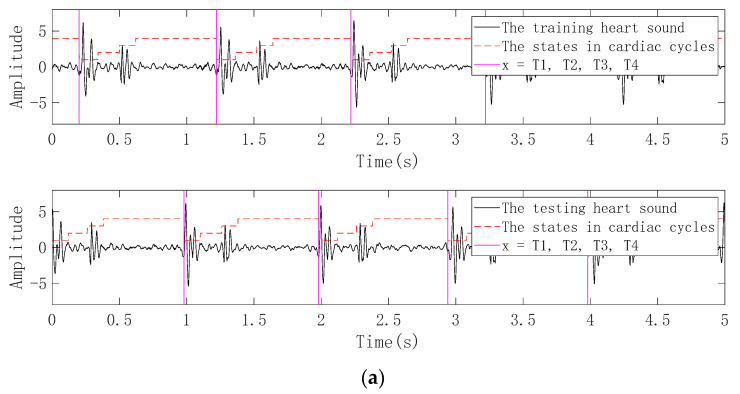

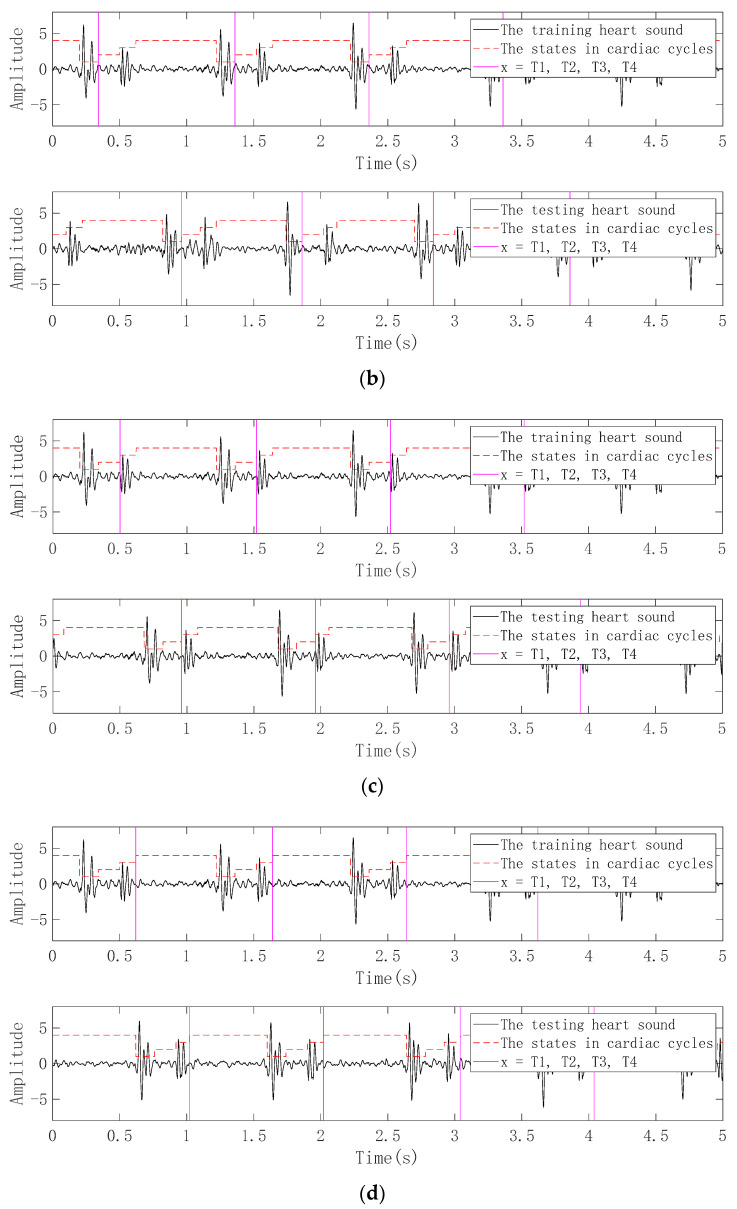
Four cardiac cycle segmentation methods based on different initial segmentation points. (**a**) The starting position of the first S1 appearing in the heart sound recording is taken as the initial dividing point; (**b**) the starting position of the first systole appearing in the heart sound recording is taken as the initial dividing point; (**c**) the starting position of the first S2 appearing in the heart sound recording is taken as the initial dividing point; (**d**) the starting position of the first diastole appearing in the heart sound recording is taken as the initial dividing point.

Although it has been considered in the previous section that the single-cycle heart sound as the test data should be aligned with the corresponding single-period heart sound in the database, the position of the initial split point is not the same when the heart sound cycle is divided. In the practical application of the heart sound biometric identification system, when the single-cycle heart sound is to be segmented from the randomly collected heart sound signal, the position of the initial segmentation point must be fixed and kept consistent. Therefore, the heart sound segmentation method based LR-HSMM proposed by Springer et al. [31] is firstly used to assign the states of the heart sound recording of 40 volunteers collected in the natural environment, and then the following four initial dividing points are used to obtain four kinds of single-cycle heart sounds as training: (1) The starting position of the first S1 appearing in the heart sound recording is taken as the initial dividing point; (2) the starting position of the first systole appearing in the heart sound recording is taken as the initial dividing point; (3) the starting position of the first S2 appearing in the heart sound recording is taken as the initial dividing point; (4) the starting position of the first diastole appearing in the heart sound recording is taken as the initial dividing point. At least one hour later, the heart sound recordings of the 40 volunteers were collected again, and four single-cycle heart sounds as testing are respectively obtained in the same manner as the single-cycle heart sounds obtained as training. A schematic diagram of four heart sound cycle segmentation methods is shown in Figure 6.

It is known from experiments that the four segments of (a), (b), (c) and (d) obtain the same number of single-cycle heart sounds: 209 single-cycle heart sounds as training and 190 single-cycle heart sounds as testing. Since the current data set is relatively balanced, the feature processing here adopts another method different from the previous one: that is, one-to-one method, the feature vectors of all single-period heart sounds as training\testing corresponding to each of the individual are averaged to obtain an average feature vector, so that each individual corresponds to only one average feature vector as the training\testing. Then, the average feature vector as training/testing obtained from (a), (b), (c) and (d) is used as the input of the ICEMDAN-RCMDE-FR-ED algorithm for verification experiments. The selected parameters of the experiment are still the selected parameters in the previous section. The experimental results in Table 6 show that the first segmentation method (a) achieves the highest CRR = 97.5%, which may be since the Springer algorithm is more accurate for S1 segmentation, so the segmentation (a) makes the training and testing features closer. The difference in CRR obtained by the four segmentation methods is between 0% and 5%, and the difference in Kappa coefficients obtained by the four segmentation methods is between 0 and 0.0513. The overall is very close, which may be due to the use of the average feature vector, which enhances the robustness of the algorithm and does not affect the result due to some bad single-cycle heart sounds. Therefore, the ICEEMDAN-RCMDE-FR-ED algorithm proposed in this paper combined with the heart sound segmentation method based on the logistic regression and hidden semi-Markov model (HSMM) has high practical application value in the field of biometric identification.

### 3.4. Comparison with Related Literature

Table 7 lists performance comparisons between the proposed study and other existing heart sound biometric work. Phua et al. [3] introduced linear frequency band cepstrum (LFBC) for heart sound feature extraction and used two classifiers of vector quantization (VQ) and Gaussian mixture model (GMM) for classification and recognition. The database used Composed of 10 users, the correct recognition rate is 96%. Fatemian et al. [6] proposed a PCG signal identification and verification system. The system is based on wavelet preprocessing, feature extraction using short-time Fourier transform (STFT), feature dimension reduction using linear discriminant analysis (LDA) and majority voting using Euclidean distance (ED) for classification. As a result, the recognition result for 21 subjects was 100%, and the equal error rate (EER) verification result was 33%. Tran et al. [7] used eight feature sets such as temporal shape, spectral shape, Mel-frequency cepstral coefficients (MFCC), linear frequency cepstral coefficients (LFCC), harmonic feature, rhythmic feature, cardiac feature, GMM-super vector as heart sound biometric recognition features, using two feature selection techniques, and using SVM for 52 users classification recognition, the first experiment achieved more than 80% accuracy and the second experiment achieved more than 90% accuracy. Jasper and Othman [32] applied wavelet transform (WT) to analyze the signals in the Time-Frequency representation, then selected Shannon energy envelogram (SSE) as the feature set, and tested the performance of the feature set in a database of 10 people with an accuracy of 98.67%. Cheng et al. [1] introduced a human feature extraction method based on an improved circular convolution (ICC) slicing algorithm combined with independent subband function (ISF). The technology uses two recognition steps to obtain different human heart sound characteristics to ensure validity, and then uses similar distances for human heart sound pattern matching. The method was verified using 10 recorded heart sounds. The results show that the two-step recognition accuracy is 85.7%. Cheng et al. [8] used heart sound linear band frequency cepstrum (HS-LBFC) for feature extraction and used similar distances for classification. The results were done on 12 heart sound signals, with a verification rate as high as 95%, false acceptance rate = 1% to 8%, and a false rejection rate of less than 3%. Zhao et al. [11] used the heart sound database of 280 samples constructed by 40 users to test their proposed marginal spectrum (MS) features and validated them using 80 samples randomly selected from the open database HSCT-11. Gautam and Deepesh [33] proposed a new method for heart sound recognition, which is based on preprocessing using a low-pass filter, using autocorrelation to detect the cardiac cycle, and segmenting S1 and S2 by windowing and thresholding. The method used WT for feature extraction and back propagation multilayer perceptron artificial neural network (BP-MLP-ANN) for classification and the accuracy rate on 10 volunteers reached 90.52%, the EER reached 9.48%. Tan et al. [34] demonstrated a new method for heart sound authentication. The pre-processing is based on low-pass filtering, and then the heart sounds are segmented using zero-crossing rate (ZCR) and short-term amplitude (STA) techniques to extract S1 and S2 sounds. Features are extracted using MFCC, and features are classified using a sparse representation classifier (SRC). Fifteen users were randomly selected, and the best effect of 85.45% can be achieved. Verma and Tanuja [35] proposed a heart sound-based biometric recognition system that uses MFCC for feature extraction and SVM for classification. They studied 30 topics with an accuracy rate of 96%. Abo Zahhad et al. [36] proposed a heart sound recognition system based on 17 subjects with an accuracy rate of 99%. Features were selected using MFCC, LFCC, bark frequency cepstral coefficients (BFCC) and discrete wavelet transform (DWT), and fused using Cone Correlation Analysis (CCA). GMM and Bayesian rules were used for classification. Abo Zahhad et al. [37] used HSCT-11 and BioSec. databases to compare the biometric performance of MFCC, LFCC, wavelet packet cepstral coefficient (WPCC) and non-linear frequency cepstral coefficients (NLFCC), and the conclusion is that WPCC and NLFCC have better biometric performance in high noise scenarios.

Compared with the above work using different feature extraction, classification methods and heart sound database, we can conclude that our method has the best effect in the same size heart sound database. The previous methods were performed on normal healthy subjects, without taking into account heart disease, and this paper conducted research on the open pathological heart sound library Michigan, Washington, and Littman. Different from the previous method, we first use the LR-HSMM-based heart sound segmentation method proposed by Springer [31] to segment the pre-processed heart sound record into a series of single-cycle heart sounds, and frame and window based on each cycle length to ensure that Each single cycle heart sound can get the same number of frames. Different from the previous method, we first introduced RCMDE features for heart sound biometrics, and selectively combined RCMDE with ICEEMDAN, FR and ED methods, and strived to improve the mixed Recognition rate of normal and pathological heart sounds with more fine characterization. The proposed method not only achieved a correct recognition rate of 96.08% on the open heart sound database, but also achieved a recognition rate of 97.5% on the 80 heart sound database composed of 40 healthy subjects constructed by the research group, and draw the conclusion that the single-cycle heart sound recognition rate from the first heart sound (S1) to the next S1 is the highest.

## 4. Conclusions

In the current research, based on the characteristics of the heart sound signal, the improved ensemble empirical mode decomposition (ICEEMDAN), fine composite multiscale dispersion entropy (RCMDE), Fisher ratio (FR) and Euclidean distance (ED) is used to study the mixed recognition of normal and pathological heart sounds, the following conclusions were reached:

(1) Given the quasi-periodic and non-steady-state characteristics of heart sound signals, this paper first uses LR-HSMM-based heart sound segmentation to divide heart sounds into a series of single-cycle heart sounds, and framing and windowing based on each cycle length to ensure each single cycle heart sound can get the same number of frames.

(2) To solve the problem of unified representation of heart sound frames with different lengths, this paper first introduces RCMDE for heart sound biometric identification and selectively combines RCMDE with ICEEMDAN, FR and ED methods for heart sound Biometric characterization.

(3) The recognition rate of this method on the open pathological heart sound database Michigan, Washington and Littman reached 96.08%, that is, the method can effectively recognize normal and pathological heart sounds.

(4) To enhance its practical application value, this paper applies the proposed method to a self-built heart sound database. Research shows that the single-cycle heart sound recognition rate from the first heart sound (S1) to the next S1 is the highest, which is 97.5%.

Although the features of this article have been proven to have a good effect on heart sound biometrics, we believe that each biometric has its limitations, and the future research direction is bound to integrate the outstanding performance features, and then use the latest powerful classifiers, such as deep learning methods, achieve optimal recognition. It is even possible to consider using a combination of feature extraction techniques for different signals, such as Abo-Zahhad et al. [38] proposed to use both ECG and PCG signals in a multimodal biometric authentication system, and Bugdol, M.D. et al. [39] proposed the multimodal biometric system combining ECG and sound signals. Of course, we also need to consider the impact of subject age, database size, race, gender and disease status on the performance of the biometric system. In the future, we can use features that are less affected by these factors to fuse or use only specific features to biometrically identify specific populations, such as using the method in this article to biometrically identify people with heart disease, so the research in this article can be used as a foundation for future biological identification research.

## Figures and Tables

**Table 1:** Comparison of the recognition performance of setting different frame length and frameshift based on the cardiac cycle on the three open heart sound databases.

Heart Sound Database	Including 2005 Single-Cycle Heart Sounds from the Open Database Michigan, Washington, and Littman		
Algorithm	RCMDE-ED		
win (inc = win)	$\bar{CRR}$	inc (win = T/4)	$\bar{CRR}$
T	45.16%	win	84.55%
T/2	78.71%	win/2	84.82%
T/3	82.52%	win/3	88.88%
T/4	84.55%	win/4	87.11%
T/5	81.09%	win/5	90.08%
T/6	82.83%	win/6	88.68%
T/7	81.56%	win/7	88.20%
T/8	76.77%	win/8	88.64%
T/9	75.13%	win/9	89.66%
T/10	65.40%	win/10	89.47%

**Table 2:** Comparison of the recognition performance of taking different intrinsic mode functions (IMFs) as the input of the algorithm on the three open heart sound databases.

Heart Sound Database	Including 2005 Single-Cycle Heart Sounds from the Open Database Michigan, Washington, and Littman		
Algorithm	ICEEMDAN-RCMDE-ED		
Input	$\bar{CRR}$	Input	$\bar{CRR}$
IMF 1	90.04%	IMF 5	41.08%
IMF 2	88.96%	IMF 6	24.28%
IMF 3	82.68%	IMF 7	14.94%
IMF 4	59.58%	IMF 8	12.15%

**Table 3:** Comparison of the recognition performance of RCMDE and ICEEMDAN-RCMDE-Fisher ratio (FR) algorithms on the three open heart sound databases.

Heart Sound Database	Including 2005 Single-Cycle Heart Sounds from the Open Database Michigan, Washington, and Littman		
Feature Extraction	Numbers of Feature	$\bar{CRR}$	Kappa Coefficients
RCMDE	320	90.08%	0.8994
ICEEMDAN-RCMDE-FR	300	96.08%	0.9602

**Table 4:** Comparison of average test accuracy μ, standard deviation σ and t value corresponding to n random trials.

*n* Random Trials	The Average Test Accuracy μ	Standard Deviation σ	*t* Value	The Critical Value Range $\left[-t_{\left(\alpha ,n\right)},t_{\left(\alpha ,n\right)}\right]$	The Degree of Confidence 1−α
*n* = 10	0.9681	0.0147	1.570	[−2.262, 2.262]	0.95
*n* = 20	0.9653	0.0146	1.378	[−2.093, 2.093]	0.95
*n* = 30	0.9634	0.0144	0.989	[−2.045, 2.045]	0.95
*n* = 50	0.9639	0.0161	1.362	[−2.010, 2.010]	0.95
*n* = 100	0.9603	0.0162	−0.309	[−1.984, 1.984]	0.95
*n* = 200	0.9608	0.0162	0	[−1.972, 1.972]	0.95
*n* = 300	0.9606	0.0162	−0.214	[−1.968, 1.968]	0.95
*n* = 400	0.9608	0.0162	0	[−1.966, 1.966]	0.95
*n* = 500	0.9607	0.0162	−0.138	[−1.965, 1.965]	0.95
*n* = 600	0.9608	0.0162	0	[−1.964, 1.964]	0.95

**Table 5:** Comparison of recognition performance of SVM, KNN and ED classifier on the three open heart sound databases.

Heart Sound Database	Including 2005 Single-Cycle Heart Sounds from the Open Database Michigan, Washington, and Littman			
Feature Extraction	ICEEMDAN-RCMDE-FR			
Classifier	Classifier Parameter	Speed	$\bar{CRR}$	Kappa Coefficients
SVM	*c* = 64, *g* = 0.001	Slowest	95.91%	0.9585
KNN	*k* = 5	Medium	73.14%	0.7276
	*k* = 3		84.83%	0.8462
	*k* = 2		95.97%	0.9591
ED and the close principle	None	Fastest	96.08%	0.9602

**Table 6:** The recognition effect of ICEEMDAN-RCMDE-FR-ED on the self-built heart sound database.

Heart Sound Database	Including the 80 Heart Sound Recordings from the Self-Built Heart Sound Database	
Algorithm	ICEEMDAN-RCMDE-FR-ED	
The Starting and Ending Position of the Input Single-Cycle Heart Sound	CRR	Kappa Coefficients
the starting position of S1—the starting position of next S1	97.5%	0.9744
the starting position of systole—the starting position of next systole	92.5%	0.9231
the starting position of S2—the starting position of next S2	95.0%	0.9487
the starting position of diastole—the starting position of next diastole	95.0%	0.9487

**Table 7:** Compared with the related literature.

Comparative Literature	Heart Sound Database	Feature Extraction	Classifier	Accuracy
Phua et al. [3]	10 people	LFBC	VQ	CRR = 94%
			GMM	CRR = 96%
Fatemian et al. [6]	21 subjects	STFT	LDA and ED	CRR = 100% EER = 33%
Tran et al. [7]	52 users	temporal shape, spectral shape, MFCC, LFCC, harmonic feature, rhythmic feature, cardiac feature and GMM-super vector	RFE-SVM	CRR = 80% CRR = 90%
Jasper and Othman [32]	10 people	WT-SSE	Template matching	CRR = 98.67%
Cheng et al. [1]	12 people 300 HS	HS-LBFC	similar distances	CRR = 99%
Cheng et al. [8]	10 people	ICC-ISF	similar distances	CRR = 85.7%
Zhao et al. [11]	40 participants 280 samples	MS	VQ and ED	CRR = 94.16%
	HSCT-11 80 subjects			CRR = 92%
Gautam and Deepesh [33]	10 subjects	segment S1 and S2 by windowing and thresholding +WT	BP-MLP-ANN	CRR = 90.52% EER = 9.48%
Tan et al. [34]	52 users	extract S1 and S2 by ZCR and STA techniques + MFCC	SRC	CRR = 85.45%
Verma and Tanuja [35]	30 people	MFCC	SVM	CRR = 96%
Abo Zahhad et al. [36]	17 subjects	MFCC, LFCC, BFCC and DWT+ CCA	GMM and Bayesian rules	CRR = 99%
Abo Zahhad et al. [37]	HSCT-11 206 subjects	WPCC	LDA and Bayesian Decision Rules	CRR = 90.26%
		NLFCC		CRR = 92.94%
	BioSec. 21 subjects	WPCC		CRR = 97.02%
		NLFCC		CRR = 98.1%
The proposed method	Michigan, Washington, and Littman 72 subjects	segment cardiac cycle by LR-HSMM + framing and windowing + ICEEMDAN-RCMDE-FR	SVM	CRR = 95.91%
			KNN	CRR = 95.97%
			ED and the close principle	CRR = 96.08%
	40 users 80 HS		ED and the close principle	CRR = 97.5%
